# Protein Nutrition for Endurance Athletes: A Metabolic Focus on Promoting Recovery and Training Adaptation

**DOI:** 10.1007/s40279-025-02203-8

**Published:** 2025-03-21

**Authors:** Oliver C. Witard, Mark Hearris, Paul T. Morgan

**Affiliations:** 1 Centre for Human and Applied Physiological Sciences, Faculty of Life Sciences and Medicine, King’s College London, Strand Campus, Strand, London, WC2R 2LS UK; 2 Department of Sport and Exercise Sciences, Institute of Sport, Manchester Metropolitan University, Manchester, UK

## Abstract

The purpose of this narrative review is to provide an evidence-based update on the protein needs of endurance athletes with a focus on high-quality metabolic studies conducted on the topics of recovery and training adaptation over the past decade. We use the term ‘protein needs’ to delineate between the concepts of a daily protein requirement and per meal protein recommendations when devising scientific evidence-based protein guidelines for the endurance athlete to promote post-exercise recovery, enhance the adaptive response to endurance training and improve endurance performance. A habitual protein intake of 1.5 g/kg of body mass (BM)^−1^·day^−1^ is typical in male and female endurance athletes. Based on findings from a series of contemporary protein requirement studies, the evidence suggests a daily protein intake of ~ 1.8 g·kgBM^−1^·day^−1^ should be advocated for endurance athletes, with the caveat that the protein requirement may be further elevated in excess of 2.0 g·kgBM^−1^·day^−1^ during periods of carbohydrate-restricted training and on rest days. Regarding protein recommendations, the current lack of metabolic studies that determine the dose response of muscle protein synthesis to protein ingestion in relation to endurance exercise makes it difficult to present definitive guidelines on optimal per meal protein intakes for endurance athletes. Moreover, there remains no compelling evidence that co-ingesting protein with carbohydrate before or during endurance exercise confers any performance advantage, nor facilitates the resynthesis of liver or muscle glycogen stores during recovery, at least when carbohydrate recommendations are met. However, recent evidence suggests a role for protein nutrition in optimising the adaptive metabolic response to endurance training under conditions of low carbohydrate and/or energy availability that represent increasingly popular periodised strategies for endurance athletes.

## Key Points

**Table:** 

The indicator amino acid oxidation method offers the most contemporary technique for estimating the protein requirements of endurance athletes and is fundamental to recent advances in informing context-specific (i.e. during carbohydrate-restricted or low energy availability training) and individualised daily protein intake guidelines during training and rest days.
Based on contemporary studies utilising the indicator amino acid oxidation method, endurance athletes require a daily protein intake of 1.8 g·kg of body mass (BM)^−1^·day^−1^, which is 50% greater than sedentary adults, but should be further elevated to ~ 2.0 g·kgBM^−1^·day^−1^ during intensive training periods conducted under conditions of carbohydrate restriction and/or low energy availability and on rest days.
Preliminary evidence indicates that endurance athletes should target a per meal protein intake of ~ 0.5 g·kgBM^−1^ to maximally stimulate the synthesis of contractile muscle proteins during immediate post-exercise recovery.
Although awareness around the under-representation of female participants in sport nutrition research is beginning to improve, experimental studies designed to inform the protein needs of female endurance athletes remains a priority gap in the knowledge that warrants investigation.

## Introduction

**Fig. 1:**
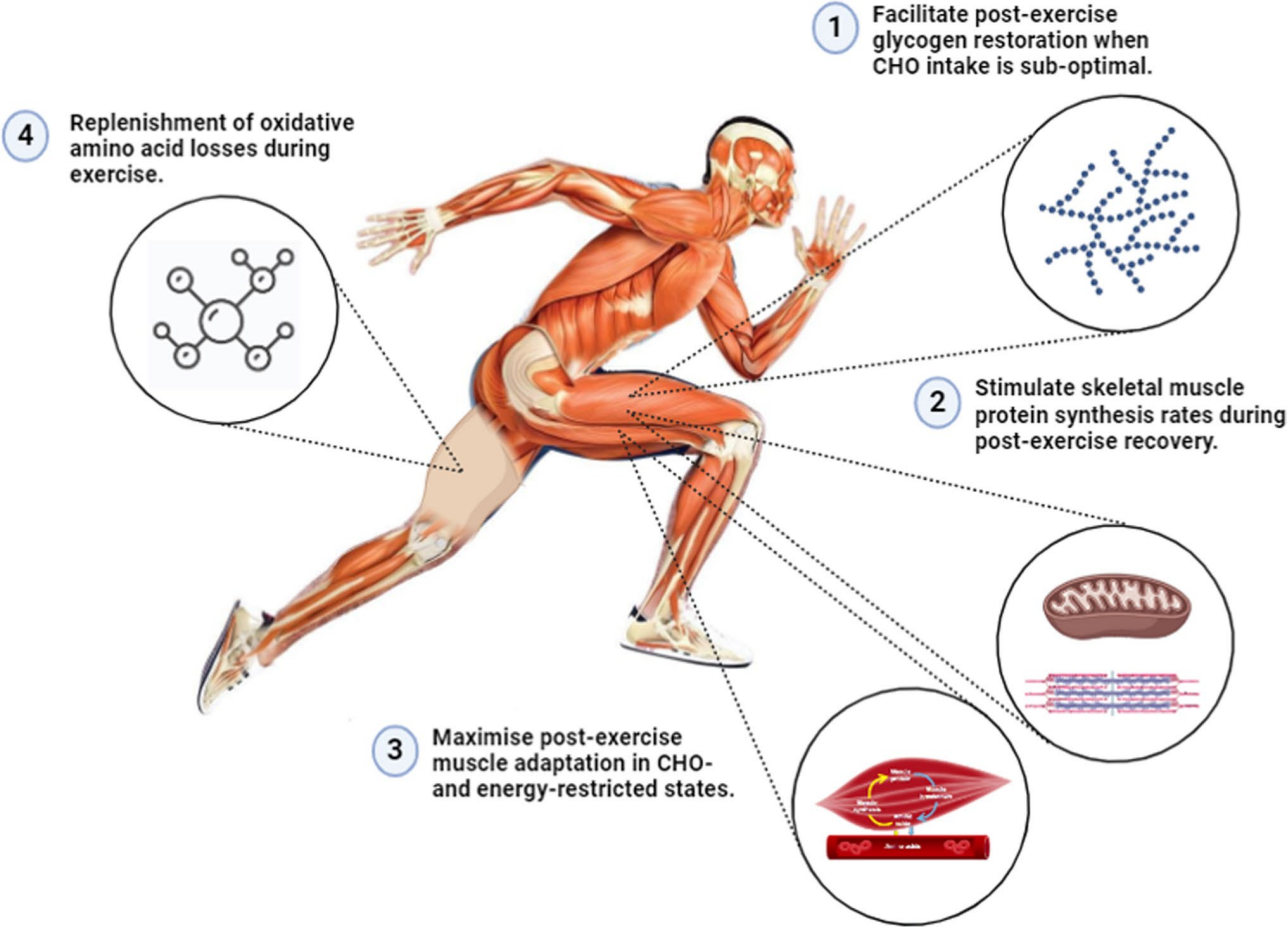
Proposed mechanisms that underpin the role of protein nutrition in optimising endurance performance by promoting training adaptations and enhancing post-exercise recovery. Dietary protein intake is suggested to be an important factor in (1) facilitating muscle glycogen resynthesis under conditions of suboptimal carbohydrate (CHO) intake, (2) stimulating muscle protein synthesis rates during recovery, (3) attenuating post-exercise protein breakdown in catabolic states such as energy restriction (e.g. fasted training) and (4) replenishing oxidative amino acid losses during exercise

The 2024 Paris Olympic and Paralympic games showcased many extraordinary feats of athletic performance with  more than 30 world records broken across an array of sports, disciplines, and events. Among other scientific disciplines in sports medicine (e.g. exercise physiology, physiotherapy, strength and conditioning, sport psychology), sport nutrition plays a crucial role in optimising athlete health, training adaptation and competitive performance [1]. Dietary protein intake is widely recognised as fundamental to maximising muscle hypertrophy and strength with resistance training in strength and/or power-based athletes [2–4]. However, despite a solid scientific rationale (Fig. 1), less emphasis has historically been placed on the role of protein nutrition in promoting post-exercise recovery, adaptations to endurance training and optimising performance in endurance athletes [5]. Hence, the purpose of this narrative review is to comprehensively update [6] and critically appraise recent advances in scientific knowledge primarily from metabolic studies regarding the protein needs of endurance athletes. The majority of relevant metabolic studies used to inform protein recommendations in endurance athletes have recruited trained cyclists and/or triathletes that conducted an acute bout of prolonged (~ 90-min) continuous exercise typically on the cycle ergometer [7, 8], with only a handful of studies conducted in other enduranced-based modalties (e.g., running), team sport (i.e. soccer, ice hockey, volleyball) athletes [9, 10] or utilising a concurrent (combination of endurance exercise and resistance exercise) exercise model [11, 12]. In addition, data generated regarding protein needs in the context of ultra-endurance events (i.e. > 5 h) is comparatively limited [13]. In a bid to bridge the gap between science and practice, where possible, we translate the findings from classic and contemporary experimental metabolic studies of protein nutrition into practical recommendations for athletes and sport nutrition professionals within the context of endurance athlete performance.

## Protein Metabolism and Endurance Exercise

The primary nutritional role of dietary protein in the context of exercise metabolism relates to the provision of amino acids as a substrate and signal for the repair and remodelling of skeletal muscle proteins. Muscle protein synthesis (MPS) and muscle protein breakdown (MPB) rates are stimulated both during and after endurance [14] or resistance [15] exercise and underpin the cumulative response of skeletal muscle remodelling to exercise training. In addition to the remodelling of contractile force-generating myofibrillar proteins, new muscle mitochondrial proteins that power muscle energetics also are synthesised in response to exercise [16, 17], with clear functional relevance to endurance performance. In trained individuals, the response of muscle remodelling to endurance exercise is mode specific [18, 19]. Accordingly, endurance exercise stimulates the synthesis of mitochondrial muscle proteins with minimal acute changes in the synthesis of myofibrillar proteins [18]. Conversely, the increased synthesis of myofibrillar proteins and mitochondrial protein has been reported in response to resistance exercise in trained [20] and untrained [18] individuals. Whilst exercise represents the most potent stimulus of MPS [21], an abundant supply of essential amino acids (EAA) is necessary to switch skeletal muscle from a catabolic state (i.e. where MPB > MPS and muscle protein is temporarily being lost) to an anabolic state (i.e. where MPS > MPB and muscle protein is temporarily being gained). Thus, dietary protein plays a crucial role in supplying the amino acid “building blocks” necessary to facilitate the repair and remodelling of “old” damaged proteins and the de novo synthesis of new functional muscle proteins in response to endurance exercise [22].

Endurance exercise also results in the oxidation of amino acids at levels equivalent to ~ 6% of the total energy cost of exercise [23], with the branched-chain amino acids, namely isoleucine, leucine and valine, preferentially oxidised over other amino acids [24]. Many studies have demonstrated that endurance exercise increases the rate of branched-chain amino acid oxidation [25–27], as mediated by an increased activation of the rate-limiting enzyme branched-chain oxo acid dehydrogenase [27]. The breakdown of muscle proteins into constituent amino acids serves as a key metabolic driver of the exercise-induced increase in amino acid oxidation. In this regard, amino acids are either locally deaminated for oxidation within the muscle mitochondria as a direct fuel source or are subsequently released from skeletal muscle and taken up by the liver for gluconeogenesis [28]. Multiple demographic and exercise/nutrition-related factors, including sex, exercise intensity or duration, carbohydrate (CHO) availability and habitual protein intake all modulate amino acid oxidation rates during exercise [6], meaning a context-specific approach to determining the protein needs of endurance athletes is necessary. For instance, leucine oxidation rates were higher in men than women during 90-min of moderate intensity (65% $\dot{V}{\text{O}}_{{2}} {\text{max}}$) cycling [25], albeit in the absence of any sex differences in skeletal muscle branched-chain oxo acid dehydrogenase content (see discussion on sex-specific requirements in Sect. 4.1). Moreover, leucine oxidation rates are increased during exercise conducted under conditions of restricted CHO availability [29] and when the habitual protein intake is high [30]. Hence, given the complex metabolic fate of amino acids in response to endurance exercise, establishing the protein needs of endurance athletes is important for optimising the repair and remodelling of muscle proteins and replacement of oxidised amino acids [31].

## Defining the Protein Needs of Endurance Athletes

The foundation of any discussion around the dietary protein needs of endurance athletes relates to clearly defining and distinguishing between the terms of a ‘protein requirement’ and a ‘protein recommendation’. The *protein requirement* may be defined as ‘the minimum daily protein intake necessary to satisfy the metabolic demands of the body which includes the maintenance of body composition’ [32], and is primarily reliant on whole-body measurements of protein metabolism [33]. In comparison, the *protein recommendation* may be defined as ‘protein strategies to optimize performance in athletes by facilitating training adaptation and/or accelerating recovery’ [34] and is primarily reliant on tissue-specific (primarily skeletal muscle) measurements of muscle metabolism. Hence, throughout this review, we use the term ‘protein needs’ to delineate between the concepts of a daily protein requirement and per meal protein recommendations when devising scientific evidence-based protein guidelines for the endurance athlete to promote post-exercise recovery, enhance the adaptive response to endurance training and improve endurance performance.

The topic of protein nutrition is continually evolving with growing interest in protein recommendations for endurance sports [6]. In this regard, and dependent on a training or competition context, protein nutrition before, during and after exercise has the potential to enhance recovery, promote training adaptations, reduce fatigue and optimize endurance performance. According to a comprehensive descriptive study of endurance athletes from rowing, swimming, ice skating, road cycling, running and ultra-endurance disciplines that utilised 24-h dietary recalls, a habitual protein intake of 1.5 g/kg of body mass (BM)^−1^·day^−1^ is typical in male and female endurance athletes [35]. This relatively high protein intake was associated with high energy intakes in male (~ 12.3 MJ/day) and female (~ 10 MJ/day) endurance athletes [35]. However, protein requirements and protein recommendations are likely highly individualized, not only to an endurance population, but to an individual within an elite athlete population. The concept of devising specific amino acid requirements (particularly the EAA), in addition to, or instead of, protein requirements, has also received attention [36]. This idea is pertinent given that dietary protein sources contain divergent amino acid profiles and endurance athletes adopt a range of diet types (e.g. omnivore, vegetarian, vegan). Moreover, the concept of amino acid requirements is ostensibly based on knowledge that the EAA content of a protein source, rather than the gross protein per se, dictates amino acid availability and the subsequent metabolic response, with implications for human health, training adaptation and endurance performance. However, the detailed discussion of amino acid requirements and recommendations is beyond the scope of this review. Below, we discuss recent advances in protein requirements for endurance athletes, followed by protein recommendations for skeletal muscle remodelling, refuelling, training adaptation and performance in the context of endurance exercise.

## Protein Requirements

Nitrogen balance methodology has traditionally been deployed to assess protein requirements [33]. However, there is long-standing and valid criticism that nitrogen balance methodology systematically underestimates the true protein requirement, particularly in athletes [37]. Indeed, it is generally accepted that recommended protein intakes for athletes should exceed the current recommended daily allowance (RDA) of ~ 0.80 g·kgBM^−1^·day^−1^, which is derived from nitrogen balance data [2, 38]. Moreover, the practical relevance of maintaining nitrogen balance for athletes aiming to optimise training, recovery and performance is generally considered somewhat limited [4, 37].

A more contemporary approach to estimating protein requirements is the minimally invasive indicator amino acid oxidation (IAAO) method [39–41], although inherent methodological limitations are also associated with this technique [42]. Utilising stable isotope tracer methodology, the basic principle of the IAAO technique relates to determining the dietary protein intake that minimizes amino acid oxidation (also referred to as oxidative catabolism) and maximizes whole-body protein synthesis rates [41]. In practice, one of the EAA (typically phenylalanine, lysine or leucine) is labelled with a stable isotope (e.g. ^13^C) and the appearance of the ^13^C label in breath carbon dioxide (i.e. ^13^CO_2_) is used an indicator of the protein requirement. In the case where the chosen EAA is deficient for protein synthesis, all other amino acids, including the ‘indicator’ amino acid, cannot be directed towards synthesis, and as a result are oxidised [41]. Therefore, a greater deficiency of one or more EAA would result in higher oxidation of the indicator amino acid, and hence a greater protein requirement to maximize protein synthesis. Once the dietary requirement is met, the oxidation of the indicator amino acid plateaus, and the resulting inflection or “breakpoint” represents the mean protein requirement [40]. In practice, graded intakes of protein are fed to athletes and a breakpoint is defined as representing the protein requirement (for technical details, see [39]). Three principal limitations are associated with the IAAO technique including, but not limited to, (i) the need for multiple experimental trials, (ii) a lack of consensus regarding the necessary length of adaptation period (5 days or more) prior to IAAO studies, particularly in athletic populations with high habitual protein intakes [43], and (iii) concerns regarding whether the amount of indicator amino acid (typically phenylalanine) fed during experiments is (a) rate limiting in identifying the breakpoint in amino acid oxidation and by extension whole-body protein synthesis or (b) an artefact reflecting phenylalanine deficiency as opposed to adequacy [42]. Regardless, whereas estimates of the protein requirement based on nitrogen balance methodology may systematically underestimate the minimum protein requirement in athletes [44–46], including endurance athletes [47], equally IAAO studies may over-estimate the protein requirement. Hence, in the context of endurance exercise, there may be more value in estimating the relative requirement, (i.e. fold difference from rest or untrained states) rather than absolute estimates of the protein requirement. Nonetheless, proponents of the IAAO technique also cite maximizing whole-body protein synthesis rates as a more physiologically relevant measure for athletes compared with achieving a nitrogen equilibrium as derived from nitrogen balance studies [48]. Hence, IAAO studies have received considerable recent attention in terms of estimating protein requirements in endurance athletes.

**Fig. 2:**
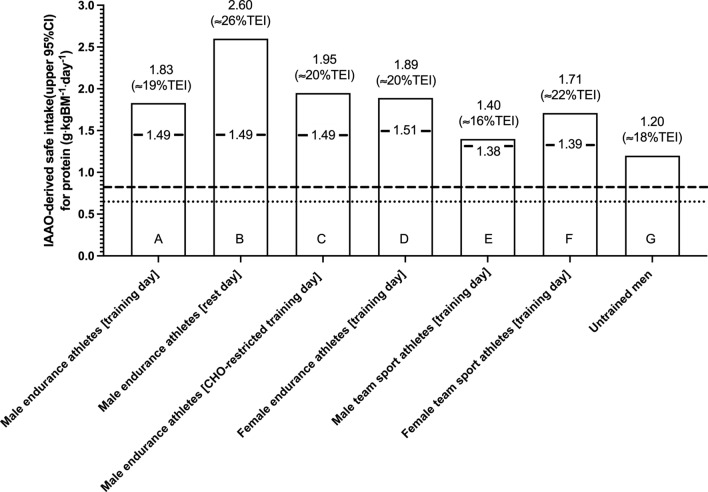
Protein requirements of endurance athletes and team sport athletes estimated using the indicator amino acid oxidation (IAAO) technique across a variety of applied settings. **A** IAAO estimate of protein requirement based on ^13^CO_2_ excretion in endurance-trained male individuals (*n* = 6) immediately following a 20-km treadmill run [47]. **B** IAAO estimate of protein requirement based on ^13^CO_2_ excretion in endurance-trained male individuals (*n* = 6) 24 h after completing a 60- to 90-min run or bike ride [49]. **C** IAAO estimate of protein requirement based on phenylalanine oxidation rates in endurance-trained male runners (*n* = 8) following a 10-km treadmill run completed in a carbohydrate-restricted or carbohydrate-fed state [50]. **D** IAAO estimate of protein requirement based on phenylalanine oxidation rates in endurance-trained female individuals (*n* = 7) during the mid-luteal phase of their menstrual cycle following a 20-km self-paced outside run [65]. **E** IAAO estimate of protein requirement based on ^13^CO_2_ excretion in male team-sport athletes (*n* = 7) immediately following a 75-min bout of intermittent exercise [9]. **F** IAAO estimate of protein requirement based on ^13^CO_2_ excretion in female team-sport athletes during the luteal phase of the menstrual cycle (*n* = 6) immediately following a 75-min bout of intermittent exercise [10]. **G** IAAO estimate of protein requirement based on ^13^CO_2_ excretion in untrained male individuals (*n* = 8) at rest [39]. *BM* body mass, *CI* confidence interval, *EAR* estimated average requirement, *IAAO* indicator amino acid oxidation, *RDA* recommended daily allowance; value inside each bar refers to an habitual protein intake (g·kgBM^−1^·day^−1^) of the corresponding category of athlete based on Gillen et al. data [35]; *estimated %TEI* percentage of total energy intake derived from protein; *dashed line* denotes current protein RDA (0.8 g·kgBM^−1^·day^−1^); *dotted line* denotes current protein EAR (0.62 g·kgBM^−1^·day^−1^)

Over the past decade, a series of carefully controlled studies, primarily conducted in the laboratory of Dr. Daniel Moore and colleagues at The University of Toronto, have utilized the IAAO technique to estimate protein requirements in endurance athletes [47, 49–51] and have revealed important context-specific conclusions (Fig. 2). A consistent finding across IAAO studies is that endurance exercise markedly increases the protein requirement on both training [47] and recovery [49, 51] days in comparison to estimated protein requirements of 1.2 g·kgBM^−1^·day^−1^ in non-active young men [39], as determined using identical methods, and also compared with habitual protein intakes of 1.5 g·kgBM^−1^·day^−1^ previously reported in endurance athletes [35]. For instance, mean protein requirements were estimated (using IAAO methodology) at 1.83 g·kgBM^−1^·day^−1^ on a training day in which endurance-trained men completed a 20-km treadmill run [47], which is 2.3-fold greater than the current RDA devised in non-athletic adult populations [33] and consistent with the notion that protein requirements are increased in response to endurance exercise [23]. This elevated protein requirement likely reflects the need to replenish the oxidative loss of branched-chain amino acids incurred during exercise [47, 52, 53] and the need to supply sufficient additional amino acids to remodel muscle proteins following high volumes of strenuous exercise training. Daily protein intakes of ~1.5 g·kgBM^−1^·day^−1^ are generally met on a habitual level in endurance athletes [35] by virtue of the higher energy demands of endurance training. However, individuals habituated to higher protein intakes may require a greater relative protein intake to account for an attenuated peripheral dietary amino acid appearance and/or enhanced amino acid oxidative capacity [54]. Interestingly, the mean protein requirement was further elevated to 1.95 g·kgBM^−1^·day^−1^ when endurance-trained men performed a similar endurance exercise bout (10-km treadmill run) under conditions of low CHO availability [50], and thus markedly exceeds previously reported habitual protein intakes in endurance athletes [35]. This finding is consistent with previous observations of higher amino acid oxidation rates during exercise that commenced in a glycogen-depleted state [29, 55], and also aligns with work indicating reduced MPS rates during energy deficit [56, 57] or applied contexts of low energy availability [58]. Moreover, and perhaps counter-intuitively, recent IAAO studies indicate that the protein requirement increases to > 2 g·kgBM^−1^· day^−1^ when measured on a passive recovery day, i.e. 24 h after endurance exercise [49, 51]. This observation is likely underpinned by the nutritional requirement to supply ample amino acids as substrate to repair and remodel damaged proteins on a whole-body level during exercise recovery [59]. Nevertheless, from a practical standpoint, this discrepancy in estimated protein requirement between training and recovery days supports the notion that a periodised approach to establishing protein requirements, both across and within a training cycle, may be beneficial for the endurance athlete. This approach is timely given the increased interest in nutritional periodization as a strategy to augment exercise-induced adaptations with endurance exercise, including training in a fasted state [60–62]. To our knowledge, no study to date has estimated protein requirements on a rest day during periods of CHO-restricted training. Finally, it is noteworthy that the metabolic demands of different endurance sports can vary, and therefore so too can protein requirements of endurance athletes [63].

### Protein Requirements of Female and Master Endurance Athletes

A notable gap in the knowledge regarding protein requirements for endurance athletes concerns the paucity of studies conducted exclusively in female athletes [64, 65] and masters athletes [66]. To our knowledge, studies conducted in female volunteers only are limited to a trio of small-scale nitrogen balance studies [25, 67, 68], including a retrospective analysis of nitrogen balance studies in endurance-trained women [68]. Regrettably, this omission reflects the distinct under-representation of women in sport and exercise medicine research [69]. Notwithstanding, a recent IAAO study was conducted in female team-sport athletes who engaged in a 75-min variable-intensity (walking, jogging, running and sprinting), intermittent exercise protocol designed to simulate team sport activity [10]. The protein requirement for female team sport athletes was calculated at 1.71 g·kgBM^−1^·day^−1^, which exceeded the estimated protein requirement of 1.40 g·kgBM^−1^·day^−1^ calculated in a comparable study of male team sport athletes that completed the same exercise protocol [9]. Interestingly, all female participants completed study trials during the luteal phase of their menstrual cycle when protein requirements may be considered highest [65]. In this regard, high oestrogen levels have been shown to decrease amino acid oxidation rates, while accelerating lipolysis and increasing fatty acid oxidation during endurance exercise [25]. Given that the mid-luteal phase is characterised by a low oestrogen:progesterone ratio, amino acid oxidation rates were likely elevated during exercise in this female cohort. In contrast, the mid-follicular phase is characterised by a high oestrogen:progesterone ratio that would likely have elicited a protein-sparing effect (in terms of amino acid oxidation) during exercise, thereby reducing the protein requirement. While the protein requirement of female athletes may theoretically be increased during the luteal phase of the menstrual cycle [64], experimental evidence is still required to substantiate the practical guideline for a periodized approach to protein requirements based on menstrual status [70]. Moreover, whereas a seminal study utilizing nitrogen balance methodology indicated that protein requirements (0.94 g·kgBM^−1^·day^−1^) are not substantially elevated in middle-aged (~ 57 years) endurance-trained men versus untrained men [71], to this end, no study has utilized IAAO methodology to determine protein requirements in older (aged > 65 years) masters endurance athletes [66] and this warrants future investigation.

## Protein Recommendations

Optimizing competition performance is fundamental to all sub-disciplines of sport nutrition, including protein recommendations. The idea that protein ingestion during prolonged exercise may improve endurance performance essentially stemmed from two experimental studies that were conducted in trained cyclists [72, 73]. Both studies concluded that ingesting a typical 7% CHO-based sports drink (26 g of CHO) with the addition of a modest 6.5 g dose of protein during exercise resulted in a ~ 30% improvement in endurance capacity compared with ingesting the CHO-containing sports drink alone [72, 73]. However, three key methodological considerations limit the practical application of these findings to elite endurance athletes. First, the CHO content of the sports drink was suboptimal for endurance performance. Second, nutritional conditions were not matched for energy content, and it was hence not possible to delineate between the effect of protein per se or additional energy provided by the protein. Indeed, when energy-matched CHO and CHO plus protein drinks were ingested at 30-min intervals during 4 h of recovery from a 90-min treadmill run, no differences in endurance capacity were observed between conditions in trained runners [74]. Finally, the exercise time to fatigue test measures endurance capacity rather than endurance performance. Accordingly, two follow-up studies addressed these issues and concluded that when trained athletes ingested a sports drink during exercise at a rate considered optimal for CHO delivery, protein elicited no additional performance benefit during a validated time trial measurement of endurance performance [75, 76]. Hence, the general consensus exists that co-ingesting protein with CHO during exercise does not improve endurance performance compared with CHO ingestion alone, at least when conditions were matched for energy content and the exercise stimulus was prolonged (i.e. 60–120 min) rather than ultra-endurance (5 h plus) in nature [77]. Follow-up studies are warranted to investigate the ergogenic potential of co-ingesting protein with CHO on ultra-endurance performance [78, 79], given previous observations of improvements in protein balance with protein plus CHO ingestion during a 6 h bout of exercise [13]. Nonetheless, this lack of effect of protein ingestion during prolonged exercise on endurance performance may explain, at least in part, the relative dearth of information historically available regarding protein recommendations for endurance athletes compared with strength or power-based athletes. Notwithstanding, more recent attention has focussed on protein recommendations for endurance athletes in the context of facilitating post-exercise recovery and promoting training adaptations. Below, we discuss recent advances in (i) protein recommendations based on the fraction-specific response of MPS to ingested protein during recovery from endurance exercise and concurrent exercise (Sects. 5.1 and 5.2); (ii) protein recommendations during CHO-restricted endurance training (Sect. 5.3) and (iii) protein recommendations to optimize glycogen restoration during post-exercise recovery (Sect. 5.4) [Fig. 1].

### Protein Ingestion and the Response of Muscle Protein Synthesis to Endurance Exercise

A limited number of studies have measured the acute response of MPS to protein ingestion and endurance exercise in comparison to equivalent studies that utilized a resistance exercise model. Early studies consistently demonstrated that protein ingestion during an acute bout of endurance exercise failed to augment MPS *during* continuous endurance exercise in trained male cyclists [22, 80], while the exercise-induced increase in MPB was attenuated with protein ingestion [22]. In contrast, protein ingestion during and/or at immediate cessation of exercise was shown to stimulate MPS [22, 81–85] and net muscle protein balance [13, 81] over the post-exercise recovery period. An intuitive explanation for this discrepant observation relates to the energetically expensive nature of MPS as a physiological process, which requires ~ 4 mol of ATP to attach (via polypeptide bonds) amino acids during the elongation phase of MPS [86]. This means that other metabolic processes, including the oxidative phosphorylation of glucose and lipids as primary substrates for ATP resynthesis, were likely prioritized over other metabolic processes (i.e. activation of downstream mTORC1 cell signalling pathways) involved in stimulating MPS during prolonged exercise when the energy status of the cell is low, as indicated by an increased activation of AMP-activated protein kinase (AMPK) during endurance exercise [57]. Conversely, as energy availability is restored during the post-exercise recovery period, MPS was stimulated in the presence of an abundant supply of amino acids with protein ingestion. Interestingly, a very high (3.6 g·kgBM^−1^·day^−1^) habitual protein intake was shown to suppress MPS under fasting conditions during endurance exercise recovery compared with moderate (at the RDA, 0.8 g·kgBM^−1^·day^−1^) or high (1.8 g·kgBM^−1^·day^−1^) habitual protein intakes in trained runners [87]. Hence, these early studies offered a useful insight into the efficacy of protein ingestion to stimulate MPS rates during endurance exercise recovery, which may be particularly relevant to endurance athletes who habitually consume a high-protein diet.

Experimental studies that measured the response of mixed MPS to protein ingestion and endurance exercise have indisputably offered valuable information into devising evidence-based protein recommendations for endurance athletes. However, owing to methodological challenges associated with measuring mitochondrial MPS rates in humans at this time [88], it could be argued that measurements of mixed MPS (i.e. the aggregate fractional synthetic rates of myofibrillar, mitochondrial and sarcoplasmic proteins) lacked specificity with regard to understanding exercise mode-specific adaptive changes in muscle, and in particular, mitochondrial protein remodelling in response to endurance exercise. To fill this gap in the knowledge, and taking advantage of timely advances in methodology, we [7] and others [8] have conducted studies to investigate the effect of protein ingestion with or without CHO on mitochondrial and myofibrillar MPS rates following endurance exercise in trained young male individuals [7]. We assigned volunteers to a protein (20 g of whey) plus CHO (50 g of glucose) condition, or a CHO-only (50 g) condition, with two drinks ingested immediately and 30-min following a prolonged (90-min) bout of intense (75% $\dot{V}{\text{O}}_{{2}} {\text{max}}$) cycling. No effect of protein ingestion on mitochondrial MPS was observed during the 4 h recovery period between conditions, whereas protein ingestion increased the stimulation of myofibrillar MPS during acute recovery from endurance exercise. A similar finding was reported within an interval training setting [89] whereby myofibrillar, but not mitochondrial protein synthesis, increased in response to nutrient provision, including protein, during exercise recovery. Taken together, and perhaps counter-intuitively, these data indicate that protein ingestion within the context of endurance exercise targets the synthesis of force-generating contractile muscle proteins over muscle proteins responsible for energy production.

The practical implications of increased myofibrillar MPS rates during acute recovery from endurance exercise with protein ingestion warrant consideration. Whereas muscle hypertrophy and/or the maintenance of structural integrity in skeletal muscle tissue may be physiologically relevant to sprint cyclists, it is possible that increased rates of myofibrillar MPS were preferentially directed towards the repair of old damaged myofibrillar proteins into their constituent amino acids [90], and the subsequent efflux of amino acids from skeletal muscle for oxidation during endurance exercise [29] rather than a muscle hypertrophic response [91, 92]. Hence, it has been speculated that the metabolic fate of ingested protein following exercise may be disproportionately directed towards replenishing the exercise-induced breakdown of myofibrillar proteins rather than mitochondrial proteins [63]. However, given that trained cyclists rather than untrained individuals were recruited in this study [7], and thus muscle damage was likely limited, the fate of ingested protein was unlikely related to the repair of damaged contractile muscle proteins. Moreover, it is conceivable that myofibrillar MPS was fibre type dependent and specific to myosin heavy chain type I muscle fibres rather than larger, more explosive type II fibres [93, 94]. Unfortunately, few studies have measured MPS at a fibre-specific level [29, 63, 90]. Nevertheless, clearly, the remodelling of non-mitochondrial (e.g. myofibrillar) proteins is considered important for optimal endurance performance.

Another methodological consideration (reviewed in [95]) may explain the lack of change in the response of mitochondrial MPS rates observed in our previous study [7], despite an increased stimulation of myofibrillar MPS with protein ingestion [96–98]. It is conceivable that the protein dose ingested in this study [7] was not sufficient to stimulate an increase in, or maximal response of, mitochondrial MPS during endurance exercise recovery. Accordingly, a recent dose–response study conducted within an endurance exercise setting revealed a greater incorporation of dietary protein-derived amino acids into de novo mitochondrial proteins following the co-ingestion of CHO (45 g dextrose plus maltodextrin) with 45 g versus 30 g of milk protein, measured over an extended 6 h recovery period in trained cyclists and triathletes [8]. Indeed, when normalised to body mass, the maximally effective protein dose for the stimulation of myofibrillar MPS following endurance exercise was calculated, based on this single study, at 0.49 g·kgBM^−1^ (95% confidence interval 0.26–0.72) [8], which actually exceeds the equivalent relative protein dose of ~ 0.24 g·kgBM^−1^ (95% confidence interval 0.18–0.30) following resistance exercise [99], although this comparison may be dependent on the duration over which MPS is measured [100]. Moreover, a 45 g casein protein bolus prior to sleep was shown to increase mitochondrial MPS rates during a 7 h overnight recovery period following endurance exercise in healthy young men [101]. Hence, it is conceivable that the 20 g dose of whey protein administered in our study [7] may have been insufficient to stimulate an increased response of mitochondrial MPS. Future work is warranted to determine the optimal protein dose for maximal stimulation of mitochondrial MPS during endurance exercise recovery [8], with a view to conducting an additional breakpoint analysis to inform relative per meal protein dose recommendations in endurance athletes.

### Protein Ingestion and Muscle Protein Synthesis During Concurrent Exercise Recovery

Concurrent training represents an adjunctive exercise modality commonly practiced by endurance athletes, whereby both resistance exercise and endurance exercise sessions are performed either as a single ‘hybrid’ training session or as a split routine between separate sessions during the same day. An acute bout of concurrent exercise has been shown to stimulate both mitochondrial and myofibrillar MPS during exercise recovery [11]. Accordingly, several acute metabolic studies have examined the efficacy of protein ingestion to potentiate the response of MPS to concurrent exercise [102] and revealed three key findings. First, and consistent with studies that utilized an endurance-only exercise model [7, 8], protein ingestion has unequivocally been shown to stimulate an increased response of mixed [80] and myofibrillar MPS [12, 103–105], but not mitochondrial MPS [12, 104, 105], during acute recovery from a single bout of concurrent exercise. This observation further supports the notion that the stimulation of mitochondrial MPS is disassociated from the requirement of exogenous amino acids to support muscle remodelling [63]. Again, the moderate dose (20–25 g) of protein ingested following concurrent exercise in these studies [12, 104, 105] may explain the failure of studies to detect an increase in mitochondrial MPS, given the recent observation that larger (30–45 g or higher) protein doses are required to stimulate mitochondrial MPS rates during endurance exercise recovery [8]. Second, substantial heterogeneity across studies appears to exist regarding the magnitude of myofibrillar MPS response to protein ingestion following concurrent exercise. For instance, whereas Camera et al. reported a 70% increase in myofibrillar MPS after protein ingestion during concurrent exercise recovery compared with placebo [12], Churchward-Venne et al. reported a 16% increase in myofibrillar MPS after protein ingestion [104]. Methodological differences between studies, including (i) participant age, sex and training status, (ii) the dose of ingested protein and (iii) study duration, likely explain these large individual variations in MPS response to protein ingestion and concurrent exercise. Finally, two recent studies observed no differences in the myofibrillar MPS response to 20 g of ingested milk protein, whey, micellar casein or leucine-enriched soy protein during acute recovery from concurrent exercise [104, 105]. Hence, the existing evidence suggests that protein ingestion, irrespective of source provided, represents an effective strategy to potentiate myofibrillar MPS rates in response to concurrent exercise [102].

### Protein Ingestion for CHO-Restricted Exercise: Implications for Endurance Training Adaptation

Another recent advance in understanding protein recommendations for endurance athletes relates to optimizing metabolic adaptation(s) to CHO-restricted training. A periodized approach to CHO nutrition whereby selected training sessions are deliberately completed with reduced CHO availability is widely regarded as a potential strategy to augment training adaptations in endurance athletes, albeit at a consequence of reduced training quality for higher intensity training sessions [106]. On a mechanistic level, CHO availability (i.e. endogenous glycogen or exogenous CHO) modulates key molecular signalling pathways that underpin metabolic adaptations to endurance training [107–109]. Hence, endurance exercise commenced in a state of low CHO availability (i.e. glycogen depleted or in the fasted state) has been shown to augment the acute molecular signalling response via activation of the AMPK-peroxisome proliferator-activated receptor-gamma coactivator-1 alpha signalling cascade that coordinates the transcription of new mitochondrial proteins associated with the endurance phenotype [107, 108]. Moreover, several studies have demonstrated that repeated exposures to low CHO availability training over a 3- to 10-week period enhanced the activity and/or content of proteins involved in oxidative metabolism (e.g. citrate synthase, β-HAD, succinate dehydrogenase, COXIV) and, in some cases, translate to improvements in exercise performance [110–113]. While these findings provide experimental evidence to support the practical application of the ‘train low’ (muscle glycogen) paradigm for endurance athletes, recent scientific interest has focussed on how the protein needs of endurance athletes should be modified during periods of CHO-restricted endurance training.

The metabolic basis for modifying the protein needs of endurance athletes during CHO-restricted training is underpinned by two key changes in amino acid metabolism during endurance exercise. In this regard, early reports of a net release of amino acids [55, 114] and marked increase in urea production [115] during an acute bout of prolonged exercise under conditions of low CHO availability were indicative of an increase in rates of MPB and a concomitant increase in the contribution of amino acid oxidation to energy expenditure [55, 114]. Accordingly, and as detailed in Sect. 4*,* a recent IAAO study concluded that endurance training with low CHO availability increased the estimated protein requirement in endurance athletes [50], albeit by a modest margin of 0.12 g⋅kgBM^−1^⋅day^−1^. However, this elevated protein requirement may be considered somewhat conservative given that, like other studies [12, 106], the study protocol consisted of a single 10-km run [50] and thus is not representative of longer-term endurance training or indeed extended competition. Although leucine oxidation rates during exercise have been shown to be only partially influenced by energy demands [116], we contend that protein requirements are likely further elevated (i.e. in excess of 0.12 g⋅kgBM^−1^⋅day^−1^) in endurance athletes completing high training volumes (equivalent to a training camp scenario) that include multiple exercise sessions performed under conditions of low CHO availability.

Preliminary evidence exists that CHO-restricted training increases daily protein requirements due, at least in part, to an increased response of MPB and oxidation of amino acids (e.g. increase in leucine oxidation and increase in alanine oxidation as a gluconeogenic precursor) during and immediately post-exercise [50]. Thus, it is intuitive that ingesting a protein or an amino acid source before, during and/or immediately after low CHO availability training may provide a practical strategy to achieve a net protein balance whilst allowing for the enhanced cell signalling responses that underpin augmented muscle adaptation(s) to endurance training. Accordingly, Taylor et al. demonstrated that ingesting a casein hydrolysate beverage before (20 g) and during (10 g) 45 min of steady-state cycling with low muscle glycogen did not impair the activation of the AMPK-peroxisome proliferator-activated receptor-gamma coactivator-1 alpha signalling cascade that mediates the augmented training response associated with low CHO availability [117]. Moreover, Impey et al. demonstrated that ingesting 22 g of whey protein before and during 120-min of steady-state cycling in the ‘fasted’ state did not alter rates of whole-body lipid oxidation or plasma non-esterified fatty acid or glycerol responses during exercise [118]. Given the potential for fatty acid availability to act as a signalling molecule involved in the regulation of the endurance phenotype [119], these data further support the notion that moderate protein ingestion before and/or during CHO-restricted training does not seem to interfere with the augmented cell signalling response during exercise. Regarding the potential for protein ingestion to attenuate increased rates of MPB under conditions of low CHO availability, recent work revealed that ingesting a 0.5 g·kgBM^−1^ bolus of whey protein hydrolysate prior to 90-min of steady-state cycling did not enhance net protein balance in non-exercising (forearm) muscle or augment protein synthesis in the exercising muscle [120]. Whilst interesting, a limitation of this study is that direct measurements of MPB and/or net protein balance in the exercising muscle were not measured, which limit our ability to interpret these data accordingly to contribute to our understanding of protein requirements for endurance athletes during low CHO availability. Nonetheless, preliminary experimental evidence exists with regard to advocating protein ingestion in close temporal proximity to endurance exercise conducted in a CHO-restricted state, and increasing the protein intake to at least 1.8 g·kgBM^−1^·day^−1^. Further work should focus on understanding the optimal doses of protein to ingest in close temporal proximity to exercise, alongside careful consideration of how protein is prescribed for endurance athletes in a low CHO/energy deficient state (e.g. protein source/quality, type, frequent feeding/distribution pattern) to support optimal adaptation, recovery and/or performance.

### Protein Ingestion to Enhance Tolerance to Intensified Training

Another practical application of protein nutrition for endurance athletes relates to improving tolerance to intensified periods of training, particularly when CHO intake is suboptimal. The impact of protein ingestion on performance and health-related outcomes within a training setting was initially studied in an occupational military context with a cohort of US Marine recruits who typically operate in a state of low energy availability [121]. This field-based study reported a substantial (~ 30%) reduction in the total number of medical visits due to bacterial or viral infections, muscle or joint problems, and heat exhaustion in military personnel who were assigned to ingest a 10 g protein supplement versus a CHO control supplement immediately after each training session during a 54-day basic military training camp. Moreover, subjective ratings of muscle soreness post-exercise were lower in the protein supplementation group on days 34 and 54 of the training camp. In a series of logical follow-up studies, and directly relevant to endurance athletes, we [122] and others [123] have reported the better maintenance of endurance performance (as determined by time trial or repeated sprint performance) following a 4- to 7-day block of intensified training when the dietary protein intake was increased up to 3 g·kgBM^−1^·day^−1^ in trained male cyclists. As a note of caution from a practical perspective, background CHO intakes ranged from 6 to 8 g·kgBM^−1^·day^−1^ in these studies, which may be considered suboptimal [122, 123] given the intense nature of the endurance training period. Moreover, these improvements in endurance performance with an increased protein intake were accompanied by favourable changes in creatine kinase levels (as a putative marker of muscle damage) [123], psychological symptoms of stress [122] and immune status [124] during intensified training, although metabolic measurements of oxidative stress, inflammation and endocrine status were not affected by the manipulation of the dietary protein intake [122, 123]. Taken together, these preliminary data indicate a potential role for protein nutrition under conditions of suboptimal CHO intake in improving tolerance to intensified periods of training, as mediated by a combination of psycho-physiological and/or immunological mechanisms. Although beyond the scope of this review, some athletes may also prefer to use protein as a means of leveraging a sustainable low-energy diet given the satiating and thermogenic effects of protein over CHO or fat, and the beneficial effects of a high-protein hypo-energetic diet in optimising body composition [125, 126], which might be considered particularly important for some endurance sports (e.g. road cycling) whereby maximizing power-to-body weight ratio is essential for performance. However, if the energy deficit is sufficiently large (i.e. > 30%), the dietary protein intake may have limited potential to mitigate lean mass loss [56]. An additional concern with high-protein low-energy diets is the trade-off with other nutrients, particularly if CHO is limiting for performance, and this notion warrants consideration when devising nutritional strategies according to an athlete’s training objectives.

### Protein Ingestion for Glycogen Restoration: Implications for Post-Exercise Refuelling

Carbohydrate serves as the primary fuel source during both prolonged and high-intensity (intermittent) endurance exercise [127], resulting in the progressive depletion of endogenous CHO stores (muscle and liver glycogen) during exercise. Given that endogenous CHO stores are typically limited to 400–600 g of glycogen, the restoration of muscle and liver glycogen is regarded as a primary determinant of recovery between successive exercise bouts and subsequent exercise capacity [128, 129]. Under conditions of adequate CHO intake, muscle glycogen can typically be restored within 24 h post-exercise [130]. However, for those athletes afforded limited time between exercise bouts, such as those competing in Grand Tours or multi-day tournaments or events, the rapid repletion of endogenous CHO stores is recognised as a key performance priority that requires targeted nutritional intervention.

Whilst CHO provides the primary substrate for replenishing muscle and liver glycogen stores, the co-ingestion of protein (and/or free amino acids) is recognized as a potential strategy to augment glycogen resynthesis rates during exercise recovery. Interestingly, the post-prandial blood glucose response to the combined ingestion of CHO and protein is markedly reduced during the post-exercise recovery period compared with the ingestion of CHO alone [131, 132]. Nonetheless, the increased insulin response with the co-ingestion of protein with CHO is proposed to increase glucose uptake into skeletal muscle and provide glucogenic amino acids for liver glycogen synthesis [133]. Studies have shown that co-ingesting protein with CHO augments muscle glycogen resynthesis under conditions when CHO is ingested at a suboptimal rate (i.e. ~ 0.8 g·kgBM^−1^·h^−1^) [134, 135]. In this context, the addition of protein and subsequent elevation in the insulin response appears to compensate for the lower amount of CHO ingested, given that rates of glycogen resynthesis have been shown to plateau at CHO intakes of 1.2 g·kgBM^−1^·h^−1^ (i.e. if the endurance athlete was to co-ingest protein with a suboptimal CHO dose, glycogen resynthesis is comparable to consuming optimal doses, e.g. 1.2 g·kgBM^−1^·h^−1^) [131]. In contrast, when adequate amounts of CHO are ingested (i.e. 1.2 g·kgBM^−1^·h^−1^), the co-ingestion of protein with CHO elicits no additional increase in muscle glycogen resynthesis during the acute post-exercise recovery period [128]. Hence, taken together, these data suggest that the addition of protein to suboptimal CHO intake may provide a practical strategy for athletes who present with a reduced appetite (thus reduced overall energy intake) and/or a reduced tolerance to a high CHO intake in the immediate post-exercise period.

The increased insulin response to co-ingesting protein with CHO may also play an important role in replenishing liver glycogen stores by promoting hepatic glucose storage [136] and providing glucogenic amino acids that can be used as precursors for glycogen synthesis [137]. To date, only one study has investigated the effect of co-ingesting protein with CHO on liver glycogen resynthesis following endurance exercise [138]. Despite markedly higher insulin levels during the post-prandial period, the addition of protein to a suboptimal CHO intake (0.8 g·kgBM^−1^·h^−1^) failed to augment liver glycogen synthesis when compared to a high CHO intake of 1.2 g·kgBM^−1^·h^−1^. To this end, no experimental study has measured liver glycogen resynthesis rates in response to co-ingesting protein with CHO under conditions of an optimal CHO intake. Moreover, from a practical standpoint it remains unknown whether the ingestion of a single bolus of protein to stimulate MPS also serves to elicit an insulin response of sufficient magnitude and duration to optimize muscle and/or liver glycogen resynthesis rates when compared to the repeated doses that have been used in previous studies (i.e. repeated protein feeding at 30-min intervals) [131, 139]. This gap in the knowledge is particularly relevant to endurance athletes given that a protein source is typically ingested within the initial phase of muscle glycogen repletion, which occurs independently of a rise in insulin levels [140].

## Conclusions

The purpose of this review was to provide an evidence-based update, with a focus on metabolic studies, on the protein needs of endurance athletes to facilitate the remodelling of mitochondrial and myofibrillar skeletal muscle proteins, accelerate the restoration of glycogen stores during post-exercise recovery, promote training adaptation(s) and improve endurance exercise performance. Table 1 provides a summary of practical guidelines regarding evidence-based protein requirements and protein recommendations for endurance athletes. In addition, we have proposed some priority research directions and experimental considerations when critically appraising the protein needs of endurance athletes. Recent advances in scientific knowledge mean that protein requirements and protein recommendations for endurance athletes can, and should, be more personalized (i.e. age, sex, body weight, habitual protein intake), context specific (i.e. exercise intensity/duration, time period between subsequent exercise bouts) and periodised (i.e. training in a state of CHO restriction of low energy availability). Future studies are required to understand the optimal daily distribution of protein intake, particularly in close proximity to exercise, with regard to maximizing mitochondrial MPS rates during endurance exercise recovery. Whilst protein ingestion may improve recovery from endurance exercise, particularly during scenarios of CHO restriction by augmenting rates of muscle glycogen restoration, it is pertinent to reiterate that CHO remains ‘king’ in terms of promoting muscle glycogen restoration and overall endurance performance.

**Table 1:** Summary of context-specific protein requirements and recommendations for endurance athletes

**Protein requirements**
1. Endurance-trained men and women should target a dietary protein intake of 1.80 g·kgBM^−1^·day^−1^ on standard training days. Female individuals in the luteal phase of their menstrual cycle may consider increasing protein intakes to 1.90 g·kgBM^−1^·day^−1^
2. Endurance athletes should target a dietary protein intake of 2.0 g·kgBM^−1^·day^−1^ on recovery days
3. Endurance athletes should target a dietary protein intake of 1.95 g·kgBM^−1^·day^−1^ on training days conducted with low CHO availability
**Protein recommendations**
1. Endurance athletes should target a protein feed of 0.5 g·kgBM^−1^ during immediate post-exercise recovery to facilitate the remodelling of contractile muscle proteins damaged in response to exercise
2. Endurance athletes should ingest a moderate (~ 10–20 g) dose of protein before and during prolonged exercise training sessions conducted in a state of low CHO availability to mitigate the exercise-induced increase MPB without impacting the muscle adaptive response to glycogen-depleted training
3. Endurance athletes that do not tolerate optimal (1.2 g·kgBM^−1^·h^−1^) CHO intakes during the immediate post-exercise recovery period should target a protein intake of 0.4 g·kgBM^−1^ to facilitate the resynthesis of muscle glycogen

*BM* body mass, *CHO* carbohydrate, *MPB* muscle protein breakdown
